# Global and regional burdens of opioid use disorder from 1990 to 2021, with future forecasts to 2050: a systematic analysis for the global burden of disease study 2021

**DOI:** 10.3389/fpubh.2025.1682094

**Published:** 2025-11-20

**Authors:** Juanzhao Cao, Ruxin Li, Guangjun Hu, Wanqiang Huang

**Affiliations:** 1 Department of Anesthesiology, Wuhan Third Hospital (Tongren Hospital of Wuhan University), Wuhan, China; 2 School of Clinical Medicine, Xi’an Medical University, Xi’an, China; 3 Department of Orthopedics, Wuhan Sixth Hospital, Wuhan, China

**Keywords:** opioid use disorder, decomposition analysis, age-period-cohort, Bayesian age-period-cohort, future forecast

## Abstract

**Introduction:**

This study comprehensively investigated the magnitude and temporal trends of the global burden of opioid use disorder (OUD) from 1990 to 2021 and predicted the disease burden in the next 29 years.

**Methods:**

The data originated from the Global Burden of Disease 2021 study. Incidence, prevalence, deaths, and disability-adjusted life years (DALYs) were analyzed by age-standardized rates. The estimated annual percentage change was calculated. The decomposition analysis was used to analyze the changes in burden globally and across the five social demographic index (SDI) regions from 1990 to 2021, with the affected population broken down into three key determinants at the group level: population aging, population growth, and epidemiological changes. Age-period-cohort analysis was used to estimate age, period, and cohort effects. Bayesian age-period-cohort modeling was used to predict the burden of OUD from 2021 to 2050.

**Results:**

In 2021, the global age-standardized prevalence rate (ASPR), age-standardized incidence rate (ASIR), age-standardized deaths rate (ASDR), and age-standardized DALYs rate of OUD were 198.489 [95% Uncertainty Interval (95%UI): 173.423-227.218], 24.544 (95%UI: 20.739-29.476), 1.194 (95%UI: 1.115-1.294), and 137.146 (95%UI: 112.293 -161.385) per 100,000 people, respectively. Among the 21 GBD regions, in 2021, High-income North America had the highest ASPR, ASIR, ASDR, and age-standardized DALYs rate of OUD. Overall, the global burden of OUD among males was significantly higher than that among females, especially in terms of deaths and DALYs. In terms of prevalent cases, globally, aging contributed 1.29%, population growth contributed 61.74%, and epidemiological changes contributed 36.97% to the increase in the burden of OUD. The global prevalence rate increased with age among people aged 20-30, decreased with age among those aged 30-80, and increased with age among people over 80. For males, the predicted ASPR, ASIR, ASDR, and age-standardized DALYs rate for OUD in 2050 are 239.62, 31.98, 2.42, and 206.44 per 100,000 people, respectively.

**Discussion:**

This study highlighted the substantial burden of OUD, particularly in High-income North America, young populations, and male populations. Population growth and epidemiological changes contributed significantly to the increase in the burden of OUD.

## 1 Background

Opioid use disorder (OUD) refers to a maladaptive pattern of opioid drug use that leads to serious harm or distress (1), including improper use of prescription opioids, using opioids as drugs, or using illegally obtained heroin (2, 3). OUD is a widespread global health problem that imposes a huge disease burden on the world. In 2019, globally, there were 21,390.4 thousand new cases of OUD, with an age-standardized incidence rate (ASIR) of 39.2 per 100,000 population; 3084.5 thousand prevalent cases, with an age-standardized prevalence rate (ASPR) of 265.9 per 100,000 population; 88.4 thousand deaths, with an age-standardized deaths rate (ASDR) of 1.1 per 100,000 populations (4). For the American population, there were 435,110 new cases of OUD, 6,607,640 prevalent cases, and 55,450 deaths (5). At the same time, the World Drug Report 2023 pointed out that in 2021, there were 39.5 million patients with drug use disorder globally, representing a 45% increase over the past 10 years.^1^ Given its high growth rate and heavy burden, it is of great necessity to pay attention to the global burden of OUD.

The Global Burden of Disease (GBD) database has the advantage of systematically analyzing and integrating global disease and health data. The use of GBD data can provide policymakers, researchers, and the public with a comprehensive understanding of the global situation of OUD (5). Therefore, this study retrieved detailed data on the latest burden of OUD from the GBD 2021 to comprehensively investigate the magnitude and temporal trends of the global burden of OUD from 1990 to 2021; and to predict the disease burden in the next 29 years. Our results contribute to the understanding of the burden of OUD and the formulation of more effective population-oriented policies and approaches.

## 2 Materials and methods

### 2.1 Data collection

The data for this study were sourced from the GBD 2021 database,^2^ which encompasses the burden of 371 diseases and 88 risk factors, covering 204 countries and regions worldwide.

### 2.2 Indicators of disease burden

We obtained the data on the burden related to OUD from 1990 to 2021, classified by age, sex, and GBD regions. The data include the incidence, prevalence, deaths, and disability-adjusted life years (DALYs), as well as the ASIR, ASPR, ASDR, and the age-standardized DALYs rate. At the same time, information on the socio-demographic index (SDI) of each state was also collected. This index is derived from three key indicators: the fertility rate of young women (under 25 years old), the education level (where the average number of years of education per individual being ≥ 15 years), and economic prosperity (the lagged income per capita). The SDI is calculated as the geometric mean of these three components, with each component being standardized to a range of 0 to 1.^3^

### 2.3 Statistical analysis

The Estimated annual percentage change (EAPC) was used to evaluate the temporal trends. If both the value of the EAPC and the lower limit of its 95% Confidence Interval (95% CI) > 0, it indicates an upward trend; while, if both the EAPC and the upper limit of its 95% CI < 0, it indicates a downward trend. Otherwise, it implies a stable trend (6).

The decomposition method proposed by Das Gupta was used to analyze the changes in burden globally and across the five SDI regions from 1990 to 2021, attributing these changes to aging, population growth, and epidemiological changes. This method can more precisely reveal how demographic and epidemiological factors influence the trends of disease burden over time (7). The Das Gupta method is an extension of the standardized processing and traditional factor decomposition methods commonly used in demography. It is used to compare differences across multiple dimensions between two groups and decompose the relative contributions of differences in different dimensions. Compared with the traditional Kitagawa factor decomposition method, the Das Gupta method focuses on solving two problems: first, it solves the problem that the interaction between different factors interferes with the decomposition results; second, it keeps other factors constant, thereby avoiding the problem that the decomposition results differ due different order of adding to the variables (8). A negative contribution value indicates that the direction of change of a factor is opposite to that of the total change, thus exerting an inhibitory or offsetting effect (9). A contribution value exceeding 100% occurs when the independent driving intensity of a certain factor is far greater than the total change rate. The core reason for this lies in the presence of interactions and counteracting effects among multiple driving factors.

The age-period-cohort (APC) model was used to analyze the changing characteristics of the global OUD burden across different age groups, time periods, and birth cohorts from 1990 to 2021. The APC model is based on the Poisson distribution and improves traditional descriptive analysis methods. It decomposes the target analysis variables from three dimensions: age, period, and cohort, thereby better analyzing the incidence, prevalence, death and DALY risks of diseases in terms of age, period, and cohort (10).

The Bayesian Age-Period-Cohort (BAPC) model was used to predict the global burden of OUD in 2050, which is a Bayesian regression method based on the age-period-cohort model. It aims to quantify the posterior distribution of parameters by introducing Bayesian inference, thereby more accurately predicting incidence rates and estimating the uncertainty of predictions (11). The BAPC model, which adopts the Integrated Nested Laplace Approximation (INLA) approach, can avoid the mixing and convergence issues that may arise from Markov Chain Monte Carlo (MCMC)-related sampling techniques. It uses a second-order random walk to smooth the priors of age, period, and cohort effects for predicting posterior deaths rates and DALYs rates (12). The calculation formula of the BAPC model is $\;\ln(\lambda_{\mathrm{ij}})=\mu +\alpha_{i}+\beta_{j}+\gamma_{k}+Z_{\mathrm{ij}}$ , where Zᵢⱼ represents the unstructured variation parameter, and $Z_{\mathrm{ij}}\sim N(0,k_{z}^{-1})$ . The BAPC analysis was conducted using the R package “BAPC.” R software (version 4.4.1) was used for statistical analysis. A *p*-value less than 0.05 indicates a statistically significant difference.

## 3 Results

### 3.1 Global and 21 GBD regions’ burden of OUD

From 1990 to 2021, the burden of OUD showed an upward trend except for ASIR (Table 1). The EAPC values for the global ASPR, ASDR, and age-standardized DALYs rate of OUD were 0.5 (95%CI: 0.33 to 0.68), 0.52 (95%CI: 0.26 to 0.77), and 0.5 (95%CI: 0.31 to 0.7), respectively, while the EAPC for the global ASIR of OUD was −0.17 (95%CI: −0.34 to −0.01) from 1990 to 2021. The global ASPR, ASDR, and age-standardized DALYs rate of OUD increased from 154.589 (95%UI: 131.062–181.259), 0.858 (95%UI: 0.764–0.927), and 103.689 (95%UI: 81.833–122.751) per 100,000 people in 1990 to 198.489 (95%UI: 173.423–227.218), 1.194 (95%UI: 1.115–1.294), and 137.146 (95%UI: 112.293–161.385) per 100,000 people in 2021, respectively. The ASIR of OUD in 2021 was 24.544 (95%UI: 20.739–29.476) per 100,000 people. In addition, in 2021, the global prevalent cases of OUD were 16,164,876 (95%UI: 14,133,120–18,431,510), incident cases were 1,942,525 (95%UI: 1,643,342–2,328,363), death cases were 99,555 (95%UI: 92,948–108,050), and DALYs were 11,218,519 (95%UI: 9,188,658–13,159,551).

**Table 1:** Opioid use disorder burden in Global, 21 GBD Regions: 1990–2021.

Location name	1990		2021		EAPC (95%CI)
	Number	ASR	Number	ASR	
Prevalence					
Global	8,120,814 (6801333–9,596,422)	154.589 (131.062–181.259)	16,164,876 (14133120–18,431,510)	198.489 (173.423–227.218)	0.5 (0.33 to 0.68)
Andean Latin America	34,735 (26316–44,688)	97.357 (75.366–121.399)	70,237 (54745–86,631)	99.225 (77.66–122.429)	0.13 (0.04 to 0.21)
Australasia	56,323 (51066–61,704)	258.021 (234.203–282.906)	89,882 (82240–98,309)	284.215 (259.185–311.84)	0.13 (−0.17 to 0.43)
Caribbean	38,656 (30319–47,885)	108.769 (87.196–132.255)	43,793 (35074–53,682)	88.279 (70.521–108.35)	−0.89 (−1 to −0.78)
Central Asia	141,487 (112834–171,149)	208.601 (170.147–248.838)	213,901 (186052–247,375)	213.648 (185.051–247.504)	−0.02 (−0.2 to 0.16)
Central Europe	94,315 (77154–112,569)	73.24 (59.502–88.018)	102,511 (89595–116,808)	89.232 (77.038–104.099)	0.6 (0.51 to 0.69)
Central Latin America	144,017 (110256–180,785)	93.432 (73.332–115.197)	235,672 (186457–288,978)	87.65 (69.372–107.418)	−0.21 (−0.31 to −0.11)
Central Sub-Saharan Africa	28,041 (21840–34,981)	63.752 (51.143–78.006)	81,132 (64601–100,053)	69.812 (56.865–83.817)	0.39 (0.36 to 0.43)
East Asia	2,438,352 (2053381–2,881,100)	189.173 (161.585–219.995)	1,533,265 (1278055–1,804,685)	94.718 (77.622–112.733)	−3.23 (−3.6 to −2.86)
Eastern Europe	945,657 (810645–1,103,066)	395.715 (340.507–465.058)	890,549 (787614–1,008,612)	431.533 (379.315–493.253)	−0.36 (−1.02 to 0.3)
Eastern Sub-Saharan Africa	86,687 (67284–108,378)	60.158 (48.441–72.941)	214,836 (171817–262,361)	60.318 (49.342–71.646)	−0.07 (−0.11 to −0.02)
High-income Asia Pacific	177,789 (139351–216,706)	94.781 (73.983–116.112)	162,536 (132441–194,091)	90.219 (71.082–109.56)	−0.1 (−0.21 to 0.02)
High-income North America	1,016,907 (865277–1,183,767)	327.387 (279.213–381.099)	6,894,161 (6086134–7,821,275)	1890.262 (1659.84–2156.244)	6.35 (5.84 to 6.86)
North Africa and Middle East	619,500 (493212–765,573)	198.337 (162.015–240.372)	1,456,242 (1241718–1,694,859)	222.338 (190.305–258.557)	0.44 (0.24 to 0.63)
Oceania	3,909 (3110–4,844)	67.036 (54.208–80.836)	9,178 (7462–11,120)	68.857 (56.554–82.59)	0.09 (0.07 to 0.11)
South Asia	909,628 (715760–1,128,916)	93.461 (76.11–112.69)	2,068,682 (1684431–2,497,346)	105.859 (87.157–126.68)	0.14 (−0.09 to 0.36)
Southern Latin America	58,849 (45275–74,009)	120.287 (92.958–150.908)	80,166 (63225–98,118)	110.896 (86.574–136.215)	−0.3 (−0.38 to −0.22)
Southern Sub-Saharan Africa	94,462 (77622–112,190)	202.065 (170.537–236.581)	110,213 (92590–129,711)	134.964 (114.303–157.308)	−1.7 (−2.08 to −1.31)
Sub-Saharan Africa	307,265 (241674–378,938)	78.583 (63.621–94.226)	659,407 (525038–806,793)	68.987 (56.378–81.717)	−0.57 (−0.7 to −0.44)
Tropical Latin America	151,744 (116450–192,062)	99.877 (77.912–123.469)	225,236 (177264–278,466)	90.817 (71.675–113.424)	−0.27 (−0.4 to −0.14)
Western Europe	751,195 (670343–844,808)	183.672 (163.199–207.132)	1,023,831 (932727–1,126,292)	237.544 (213.941–263.021)	0.42 (0.13 to 0.7)
Western Sub-Saharan Africa	98,076 (74943–123,315)	62.788 (49.536–76.873)	253,227 (198684–317,067)	61.746 (49.739–74.576)	−0.03 (−0.08 to 0.02)
Incidence					
Global	1,301,551 (1077634–1,598,053)	23.368 (19.579–28.485)	1,942,525 (1643342–2,328,363)	24.544 (20.739–29.476)	−0.17 (−0.34 to −0.01)
Andean Latin America	6,715 (5158–8,555)	17.008 (13.238–21.366)	12,380 (9823–15,471)	17.393 (13.8–21.668)	0.14 (0.06 to 0.21)
Australasia	9,220 (7965–10,662)	43.423 (37.552–50.015)	12,977 (11089–15,009)	44.874 (38.675–51.988)	−0.34 (−0.74 to 0.06)
Caribbean	6,948 (5503–8,823)	18.063 (14.448–22.328)	7,627 (6096–9,437)	15.611 (12.482–19.259)	−0.59 (−0.67 to −0.51)
Central Asia	25,909 (20775–31,992)	36.342 (29.678–44.449)	35,075 (29584–41,820)	36.681 (30.957–43.703)	−0.07 (−0.21 to 0.08)
Central Europe	16,514 (13678–19,962)	13.274 (10.825–16.187)	16,499 (14141–19,429)	16.097 (13.619–18.962)	0.55 (0.43 to 0.67)
Central Latin America	27,557 (21406–35,170)	16.055 (12.776–20.09)	40,991 (32758–50,725)	15.25 (12.191–18.855)	−0.17 (−0.26 to −0.08)
Central Sub-Saharan Africa	5,589 (4362–7,057)	11.408 (9.212–14.114)	16,045 (12748–20,234)	12.486 (10.245–15.369)	0.38 (0.35 to 0.42)
East Asia	419,882 (348059–515,521)	30.328 (25.635–36.403)	244,998 (202876–293,577)	16.715 (13.882–20.269)	−2.82 (−3.16 to −2.49)
Eastern Europe	152,974 (127089–183,616)	69.603 (57.809–83.761)	129,175 (110164–153,540)	73.317 (61.904–87.262)	−0.57 (−1.19 to 0.05)
Eastern Sub-Saharan Africa	17,387 (13745–22,221)	10.738 (8.779–13.171)	42,755 (34332–53,621)	10.767 (8.936–12.989)	−0.05 (−0.09 to −0.02)
High-income Asia Pacific	27,980 (22476–34,512)	15.145 (12.063–18.879)	23,885 (19434–28,719)	14.923 (12.047–18.419)	−0.02 (−0.12 to 0.08)
High-income North America	86,864 (71332–108,021)	30.183 (24.834–37.664)	456,337 (382680–549,886)	144.237 (120.133–174.948)	5.72 (5.13 to 6.32)
North Africa and Middle East	121,490 (94834–154,340)	34.771 (28.117–42.993)	245,271 (203638–296,380)	37.823 (31.504–45.62)	0.3 (0.14 to 0.46)
Oceania	767 (608–973)	12.059 (9.89–14.802)	1737 (1407–2,155)	12.49 (10.205–15.234)	0.11 (0.1 to 0.12)
South Asia	175,003 (140846–221,257)	16.72 (13.849–20.688)	378,428 (309715–466,046)	18.902 (15.728–23.122)	0.13 (−0.09 to 0.35)
Southern Latin America	8,960 (6918–11,184)	17.879 (13.945–22.26)	12,389 (9946–15,253)	17.754 (14.109–22.099)	−0.12 (−0.2 to −0.03)
Southern Sub-Saharan Africa	17,180 (14051–21,388)	32.94 (27.394–39.764)	19,234 (16064–23,406)	23.315 (19.68–28.097)	−1.44 (−1.76 to −1.12)
Sub-Saharan Africa	59,261 (47391–74,613)	13.554 (11.179–16.556)	127,877 (103347–160,286)	12.077 (10.046–14.669)	−0.49 (−0.59 to −0.39)
Tropical Latin America	27,857 (21478–35,429)	17.08 (13.358–21.508)	37,570 (29895–46,843)	15.816 (12.435–19.738)	−0.22 (−0.33 to −0.11)
Western Europe	84,153 (71514–99,715)	21.624 (18.272–25.732)	90,782 (78907–104,671)	24.072 (20.688–28.087)	−0.27 (−0.58 to 0.04)
Western Sub-Saharan Africa	19,106 (15024–24,340)	11.044 (8.907–13.63)	49,843 (39459–63,152)	10.883 (8.951–13.308)	−0.03 (−0.07 to 0.01)
Death					
Global	41,567 (36923–45,060)	0.858 (0.764–0.927)	99,555 (92948–108,050)	1.194 (1.115–1.294)	0.52 (0.26 to 0.77)
Andean Latin America	21 (17–26)	0.076 (0.062–0.096)	77 (61–101)	0.119 (0.094–0.156)	1.73 (1.36 to 2.1)
Australasia	331 (308–358)	1.516 (1.405–1.637)	582 (508–669)	1.736 (1.515–1.989)	−1.02 (−1.84 to −0.19)
Caribbean	22 (20–25)	0.071 (0.063–0.079)	46 (37–56)	0.092 (0.072–0.111)	−2.69 (−4.17 to −1.19)
Central Asia	224 (191–263)	0.378 (0.32–0.446)	603 (497–710)	0.631 (0.524–0.741)	1.73 (0.75 to 2.72)
Central Europe	598 (539–664)	0.448 (0.404–0.499)	779 (715–846)	0.561 (0.515–0.61)	0.35 (0.12 to 0.58)
Central Latin America	125 (117–133)	0.102 (0.095–0.108)	239 (206–277)	0.089 (0.077–0.104)	−0.85 (−1.1 to −0.59)
Central Sub-Saharan Africa	111 (65–165)	0.3 (0.174–0.443)	360 (203–548)	0.354 (0.199–0.535)	0.58 (0.37 to 0.79)
East Asia	19,318 (15372–22,653)	1.682 (1.354–1.953)	6,020 (4808–7,354)	0.329 (0.265–0.399)	−6.98 (−7.76 to −6.18)
Eastern Europe	4,451 (4119–4,823)	1.779 (1.645–1.928)	6,016 (5462–6,599)	2.553 (2.328–2.81)	0.32 (−0.7 to 1.36)
Eastern Sub-Saharan Africa	550 (380–771)	0.555 (0.38–0.772)	1785 (1211–2,305)	0.621 (0.431–0.792)	0.21 (0.13 to 0.29)
High-income Asia Pacific	140 (132–149)	0.073 (0.068–0.077)	293 (270–314)	0.123 (0.115–0.13)	0.76 (−0.09 to 1.61)
High-income North America	4,550 (4271–4,840)	1.43 (1.343–1.523)	58,205 (51549–65,872)	14.504 (12.923–16.299)	7.98 (7.74 to 8.22)
North Africa and Middle East	2021 (1652–2,405)	0.798 (0.655–0.951)	4,903 (4140–5,667)	0.808 (0.684–0.929)	0.08 (−0.13 to 0.29)
Oceania	10 (6–13)	0.189 (0.12–0.256)	15 (10–20)	0.126 (0.089–0.171)	−1.64 (−1.92 to −1.36)
South Asia	3,484 (3011–3,938)	0.463 (0.401–0.528)	8,064 (6706–9,346)	0.476 (0.399–0.55)	−0.27 (−0.47 to −0.07)
Southern Latin America	23 (21–25)	0.048 (0.044–0.053)	107 (95–123)	0.137 (0.121–0.158)	4.5 (3.89 to 5.11)
Southern Sub-Saharan Africa	352 (299–396)	0.921 (0.775–1.04)	619 (549–704)	0.879 (0.78–0.994)	−0.22 (−0.64 to 0.2)
Sub-Saharan Africa	1,084 (822–1,375)	0.376 (0.281–0.473)	2,883 (2129–3,584)	0.386 (0.292–0.469)	−0.01 (−0.11 to 0.09)
Tropical Latin America	11 (11–12)	0.009 (0.009–0.01)	87 (79–95)	0.034 (0.031–0.037)	5.08 (4.34 to 5.83)
Western Europe	4,393 (4257–4,528)	1.062 (1.028–1.095)	8,950 (8426–9,403)	1.668 (1.585–1.744)	0.73 (0.47 to 0.99)
Western Sub-Saharan Africa	71 (51–92)	0.072 (0.051–0.093)	119 (79–153)	0.052 (0.038–0.066)	−0.62 (−1.24 to 0.01)
DALYs					
Global	5,415,249 (4242001–6,437,812)	103.689 (81.833–122.751)	11,218,519 (9188658–13,159,551)	137.146 (112.293–161.385)	0.5 (0.31 to 0.7)
Andean Latin America	15,493 (10234–21,241)	43.691 (29.09–59.236)	32,556 (22900–43,839)	46.208 (32.591–61.985)	0.28 (0.18 to 0.39)
Australasia	42,362 (35245–49,108)	194.369 (161.718–225.024)	65,325 (53570–76,294)	205.477 (168.058–240.256)	−0.58 (−1 to −0.16)
Caribbean	17,184 (11461–23,286)	48.495 (32.7–64.367)	20,232 (14015–26,535)	40.772 (28.154–53.518)	−1.2 (−1.49 to −0.9)
Central Asia	70,350 (50799–90,742)	104.535 (75.93–132.927)	117,883 (89535–144,813)	117.719 (89.492–144.383)	0.35 (−0.06 to 0.76)
Central Europe	67,672 (53454–80,857)	52.446 (41.211–63.157)	74,953 (62010–87,477)	64.409 (53.158–75.799)	0.49 (0.41 to 0.58)
Central Latin America	66,016 (43818–88,786)	43.155 (29.441–56.568)	108,440 (75532–142,295)	40.333 (28.118–52.897)	−0.25 (−0.33 to −0.18)
Central Sub-Saharan Africa	17,085 (12481–22,414)	39.446 (28.798–51.845)	52,140 (37994–67,999)	45.144 (32.464–58.017)	0.52 (0.44 to 0.61)
East Asia	1,959,218 (1530332–2,320,474)	153.514 (120.428–181.165)	887,518 (681704–1,086,678)	54.368 (40.973–67.471)	−4.63 (−5.09 to −4.17)
Eastern Europe	600,805 (472699–720,761)	250.748 (195.861–302.22)	657,685 (555403–766,236)	311.153 (259.014–365.832)	−0.01 (−0.85 to 0.84)
Eastern Sub-Saharan Africa	61,834 (46368–79,614)	46.11 (34.978–59.655)	177,706 (136841–219,977)	51.62 (40.038–62.98)	0.29 (0.27 to 0.31)
High-income Asia Pacific	80,967 (54616–106,226)	43.181 (29.207–57.205)	78,890 (55978–100,983)	43.743 (30.904–57.084)	−0.01 (−0.2 to 0.19)
High-income North America	648,336 (515178–778,216)	207.646 (165.196–248.948)	5,570,171 (4605348–6,442,015)	1502.443 (1235.96–1740.096)	7.06 (6.81 to 7.31)
North Africa and Middle East	360,787 (268099–461,099)	118.346 (89.708–146.665)	842,161 (650328–1,031,714)	128.78 (99.595–157.456)	0.34 (0.14 to 0.54)
Oceania	2,169 (1572–2,831)	36.623 (26.832–47.439)	4,603 (3268–6,104)	34.289 (24.392–44.642)	−0.27 (−0.31 to −0.23)
South Asia	531,402 (395554–661,800)	55.716 (42.261–68.098)	1,190,532 (891410–1,478,435)	61.711 (46.68–75.918)	0.03 (−0.17 to 0.23)
Southern Latin America	25,409 (16200–34,157)	51.921 (33.116–69.72)	37,409 (26191–48,811)	51.703 (36.071–67.748)	0.02 (−0.07 to 0.1)
Southern Sub-Saharan Africa	56,153 (42619–69,333)	121.872 (93.862–147.216)	71,749 (57388–85,223)	88.952 (71.724–105.111)	−1.32 (−1.71 to −0.94)
Sub-Saharan Africa	178,110 (134613–224,972)	47.052 (35.992–58.886)	411,323 (312586–507,373)	44.182 (33.973–53.728)	−0.32 (−0.41 to −0.22)
Tropical Latin America	62,921 (40534–86,421)	41.291 (27.155–55.965)	95,395 (63997–126,549)	38.506 (25.718–51.571)	−0.15 (−0.27 to −0.04)
Western Europe	551,605 (460042–645,176)	136.186 (113.604–159.534)	778,892 (657867–897,779)	178.117 (149.993–207.304)	0.25 (0 to 0.51)
Western Sub-Saharan Africa	43,039 (28939–58,125)	27.725 (18.955–36.238)	109,727 (72681–147,988)	26.841 (18.222–35.298)	−0.03 (−0.07 to 0.02)

GBD, Global Burden of Disease; DALYs, disability-adjusted life years.

Among the 21 GBD regions, in 2021, High-income North America had the highest ASPR, ASIR, ASDR, and age-standardized DALYs rate of OUD, with the age-standardized rates (ASRs) of 1,890.262 (95%UI: 1,659.84–2,156.244), 144.237 (95%UI: 120.133–174.948), 14.504 (95%UI: 12.923–16.299), and 1,502.443 (95%UI: 1,235.96–1,740.096) per 100,000 people, respectively. Eastern Sub-Saharan Africa had the lowest ASPR and ASIR of OUD, with the ASRs of 60.318 (95%UI: 49.342–71.646) and 10.767 (95%UI: 8.936–12.989) per 100,000 people in 2021, respectively. Meanwhile, Tropical Latin America and Western Sub-Saharan Africa had the lowest ASDR and age-standardized DALYs rate of OUD, respectively. From 1990 to 2021, among the 21 GBD regions, High-income North America experienced the highest increase in the ASPR, ASIR, ASDR, and age-standardized DALYs rate, with the EAPCs of 6.35 (95%CI: 5.84 to 6.86), 5.72 (95%CI: 5.13 to 6.32), 7.98 (95%CI: 7.74 to 8.22), and 7.06 (95%CI: 6.81 to 7.31), respectively, while East Asia experienced the highest decrease in the ASPR, ASIR, ASDR, and age-standardized DALYs rate, with the EAPCs of −3.23 (95%CI: −3.6 to −2.86), −2.82 (95%CI: −3.16 to −2.49), −6.98 (95%CI: −7.76 to −6.18), and −4.63 (95%CI: −5.09 to −4.17), respectively.

### 3.2 Age and gender pattern of OUD burden

**Figure 1:**
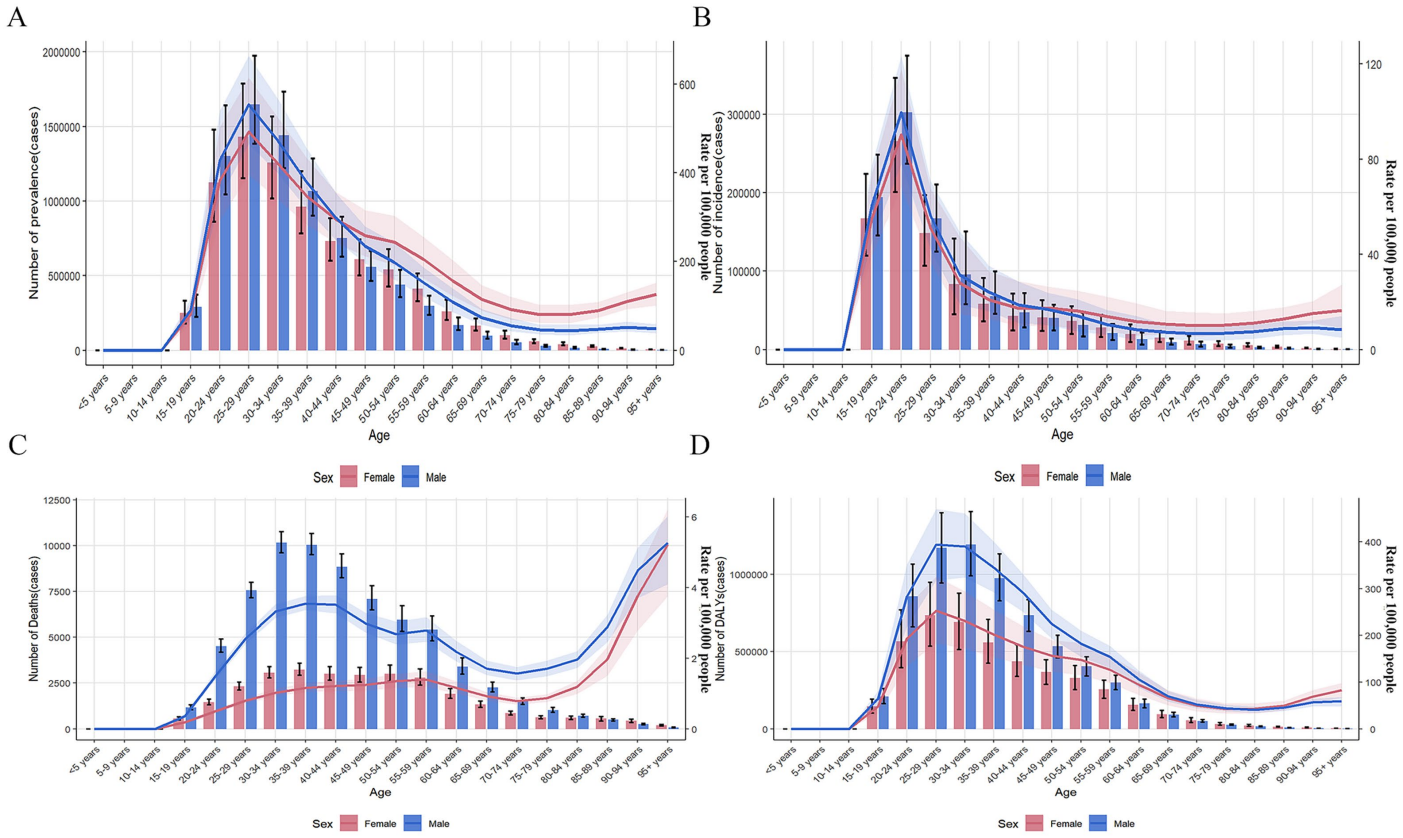
Burden of opioid use disorder by age group in global. **(A)** Prevalence, **(B)** Incidence, **(C)** Death, **(D)** DALYs. DALYs: disability-adjusted life years.

Overall, the global burden of OUD among males was significantly higher than that among females, especially in terms of deaths and DALYs (Figures 1A–D).

Regardless of gender, the number of prevalence, DALYs, ASPR, and age-standardized DALYs rate of OUD increased with age before 29 years old and decreased with age after 29 years old (Figures 1A,D). The number of incidence and ASIR of OUD increased with age before 24 years old and decreased with age after 24 years old (Figure 1B). The number of female OUD deaths increased with age before 39 years old and decreased with age after 39 years old, while the ASDR of female OUD showed an upward trend (Figure 1C). The number of male OUD deaths increased with age before 34 years old and decreased with age after 34 years old; the ASDR of male OUD showed an upward trend before 44 years old, a downward trend between 45 and 74 years old, and increased with age after 74 years old.

### 3.3 Decomposition analysis of OUD burden

**Figure 2:**
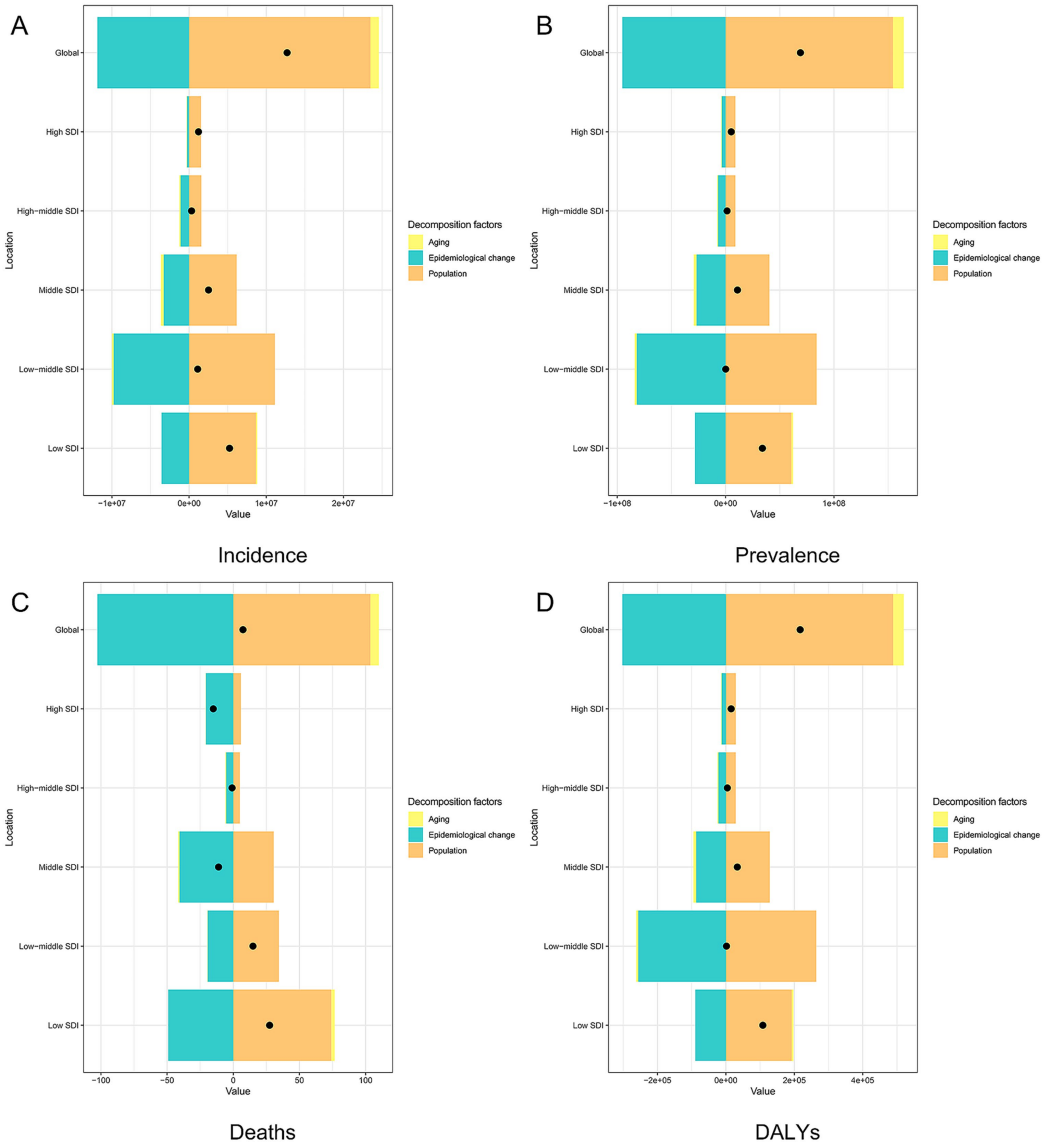
Decomposition analysis of opioid use disorder burden in global and five SDI regions. **(A)** Incidence, **(B)** Prevalence, **(C)** Death, **(D)** DALYs. SDI: social demographic index, DALYs: disability-adjusted life years.

In terms of incident cases, globally, population growth contributed 91.37% and epidemiological changes contributed 9.73% to the increase in the burden of OUD. Aging showed a positive contribution only in the high SDI regions (4.79%). Population growth had the largest positive contribution in the middle SDI regions (444.36%) and the largest negative contribution in the high-middle SDI regions (−686.41%). Epidemiological changes had the largest contribution in the high-middle SDI regions (787.75%) (Figure 2A; Supplementary Table 1).

In terms of prevalent cases, globally, aging contributed 1.29%, population growth contributed 61.74%, and epidemiological changes contributed 36.97% to the increase in the burden of OUD. The contribution of aging varied across different SDI regions: low-middle SDI regions showed a negative contribution of −11.41%, while high-middle SDI regions showed a positive contribution of 23.05%. Population growth had the largest negative contribution in the middle SDI regions (−715.37%) and the largest positive contribution in the low-middle SDI regions (99.29%). Epidemiological changes consistently showed positive contributions in all five SDI regions, with the largest contribution in the high-middle SDI regions (288.7%) (Figure 2B; Supplementary Table 1).

In terms of deaths cases, globally, aging contributed 4.23%, population growth contributed 51.86%, and epidemiological changes contributed 43.91% to the increase in the burden of OUD. The contribution of aging varied across different SDI regions: middle SDI regions showed a negative contribution of −0.96%, while high-middle SDI regions showed a positive contribution of 7.98%. Population growth had the largest negative contribution in the middle SDI regions (−111.15%) and the largest positive contribution in the low SDI regions (80.54%). Epidemiological changes consistently showed positive contributions in all five SDI regions, with the largest contribution in the high-middle SDI regions (203.17%) (Figure 2C; Supplementary Table 1).

In terms of DALYs, globally, aging contributed 1.53%, population growth contributed 58.02, and epidemiological changes contributed 40.45% to the increase in the burden of OUD. Aging showed a negative contribution only in the low-middle SDI regions (−9.19%). Population growth had the largest negative contribution in the middle SDI regions (−137.17%) and the largest positive contribution in the low-middle SDI regions (94.81%). Epidemiological changes consistently showed positive contributions in all five SDI regions, with the largest contribution in the high-middle SDI regions (222.62%) (Figure 2D; Supplementary Table 1).

### 3.4 APC model of OUD

**Figure 3:**
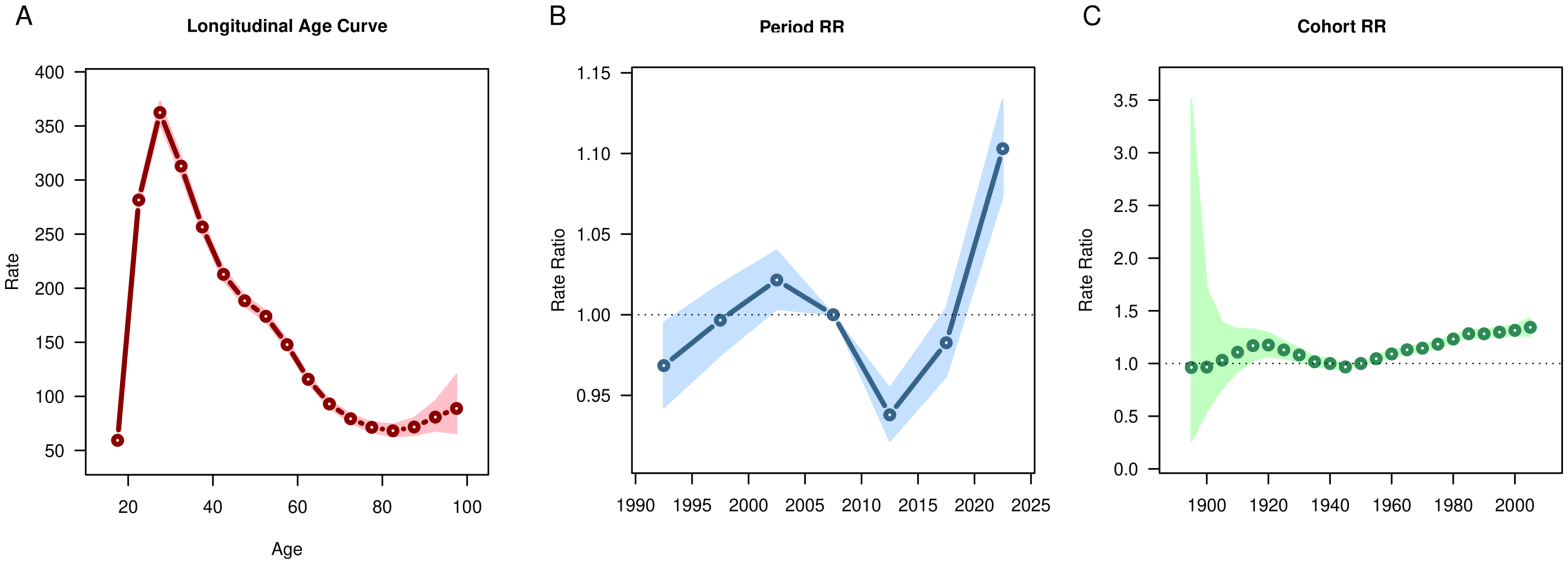
Age, period, cohort effects on opioid use disorder prevalence in global during 1990–2021. **(A)** Age effect, **(B)** Period effect; **(C)** Cohort effect.

**Figure 4:**
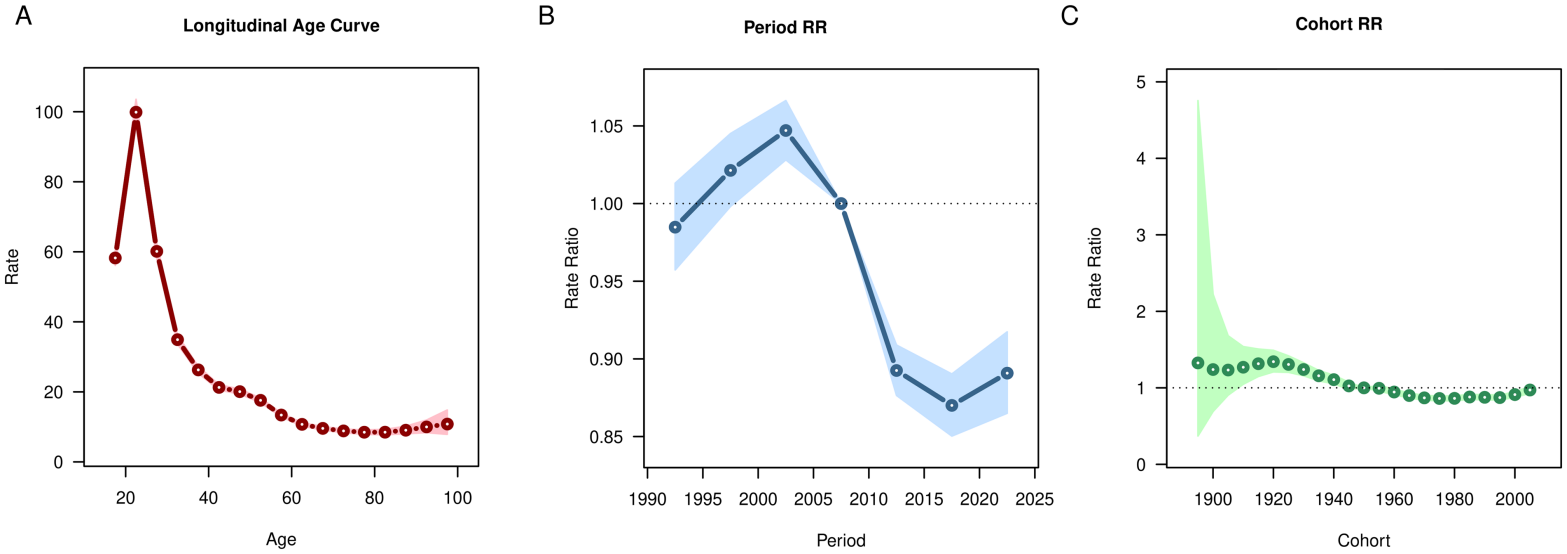
Age, period, cohort effects on opioid use disorder incidence in global during 1990–2021. **(A)** Age effect, **(B)** Period effect; **(C)** Cohort effect.

**Figure 5:**
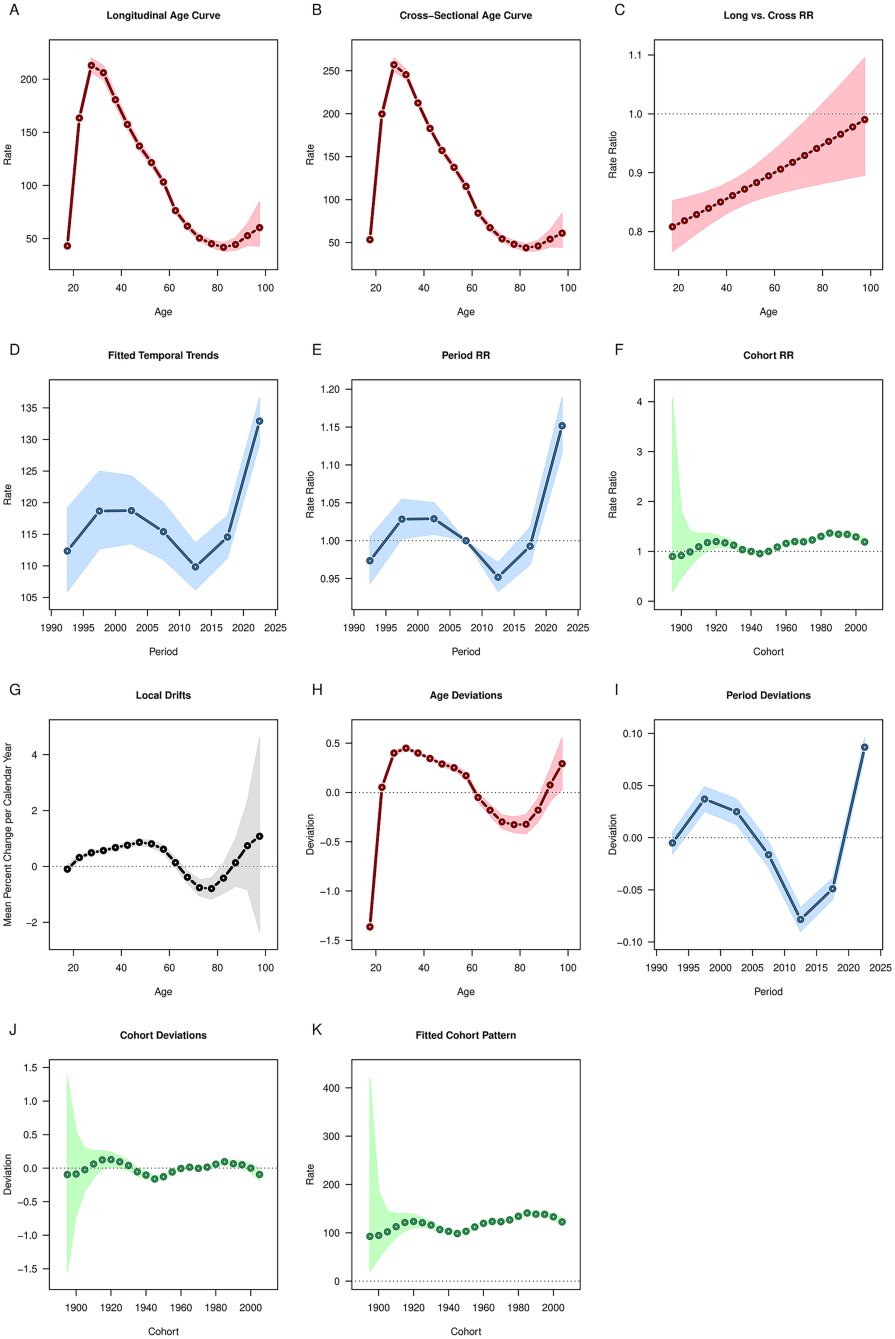
Age, period, cohort effects on opioid use disorder DALYs in global during 1990–2021. **(A)** Age effect, **(B)** Period effect; **(C)** Cohort effect. DALYs: disability-adjusted life years.

**Figure 6:**
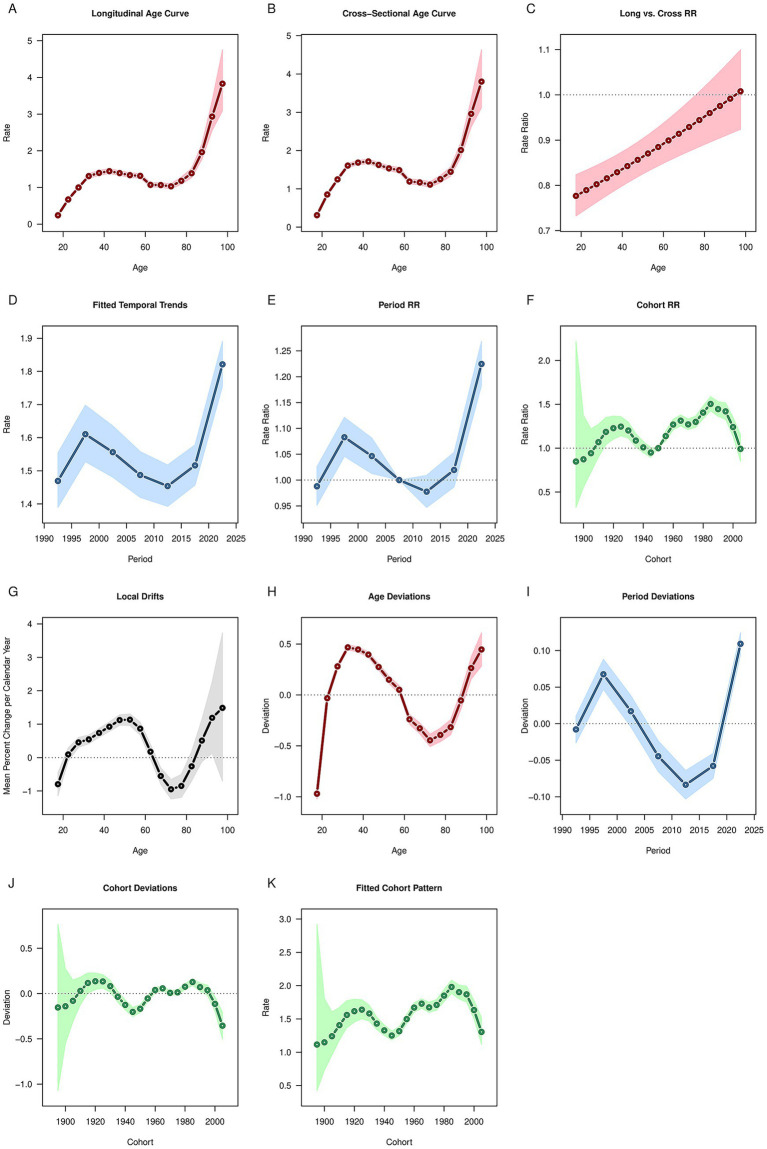
Age, period, cohort effects on opioid use disorder deaths in global during 1990–2021. **(A)** Age effect, **(B)** Period effect; **(C)** Cohort effect.

After controlling for period and cohort effects, the results of age effects showed that the global prevalence, incidence and DALYs rates of OUD exhibited a trend of first increasing, then decreasing, and then increasing again (Figures 3A, 4A, 5A). It increased with age among people aged 20–30, decreased with age among those aged 30–80, and increased with age among people over 80. The deaths rate of OUD increased with age among people aged 20–30, remained stable among those aged 30–80, and increased with age among people over 80 (Figure 6A). Globally, the period rate ratio (RR) for OUD prevalence rate and DALYs rate showed an upward trend in cohorts born before 2000–2005, a downward trend with increasing birth years in cohorts born after 2000–2005, and an upward trend with increasing birth years in cohorts born after 2010–2015 (Figures 3B, 5B). The period RR of incidence rate showed an upward trend in cohorts born before 2000–2005, then a downward trend, and another upward trend in cohorts born after 2015–2020 (Figure 4B). The period RR of deaths rate showed an upward trend in cohorts born before 1995–2000, then a downward trend, and another upward trend in cohorts born after 2010–2015 (Figure 6B). After controlling for age and period effects, the cohort RR for OUD prevalence rate and incidence rate both showed an upward trend (Figures 3C, 4C), while those for mortality and DALYs showed a downward trend (Figures 6C, 5C).

### 3.5 Future prediction of OUD burden

**Figure 7:**
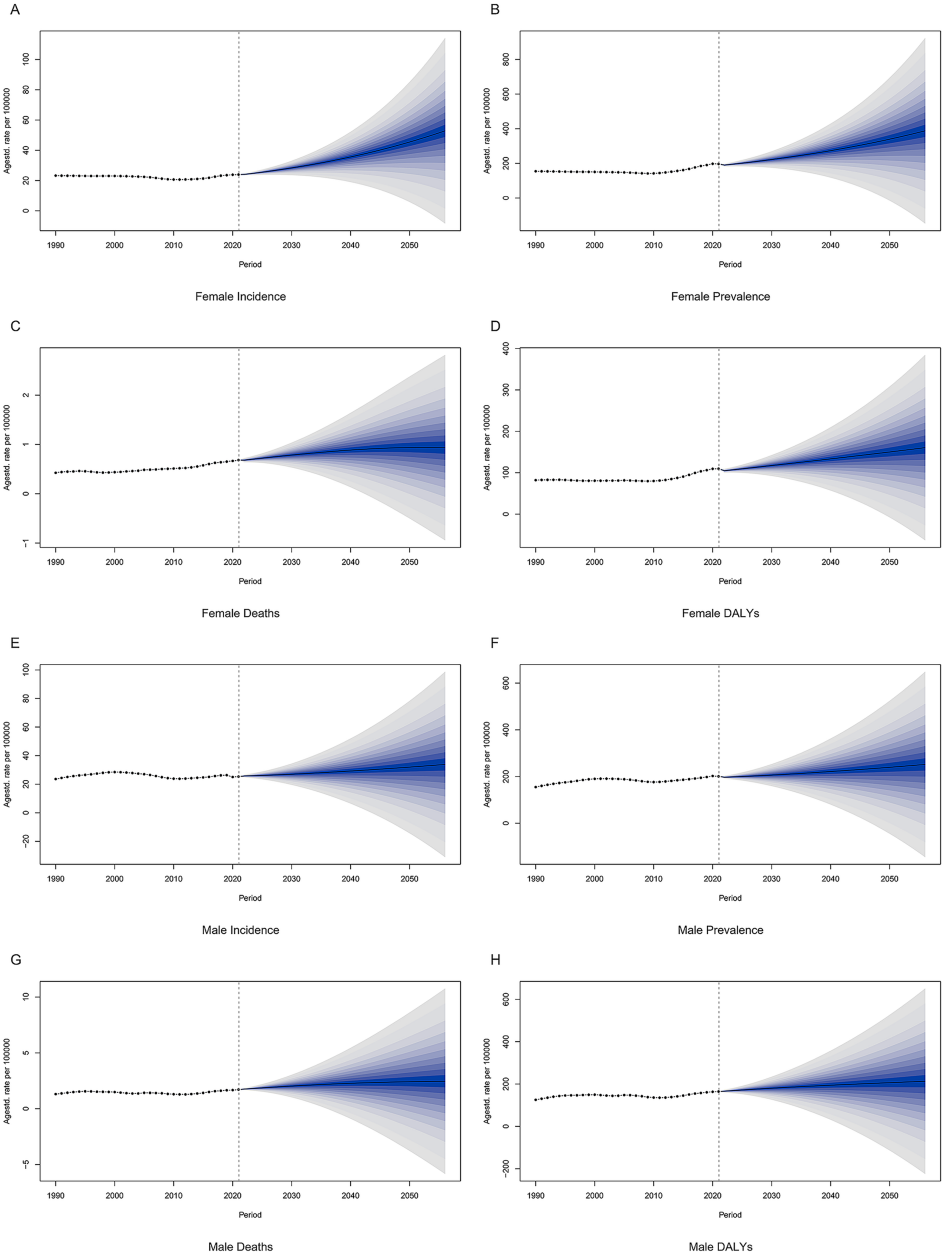
Projections of global opioid use disorder burden in the future. **(A)** Female incidence, **(B)** Female prevalence, **(C)** Female deaths, **(D)** Female DALYs, **(E)** Male incidence, **(F)** Male prevalence, **(G)** Male deaths, **(H)** Male DALYs. DALYs: disability-adjusted life years.

Regardless of gender, the future global burden of OUD shows a steady upward trend (Figure 7). Among females, the ASPR, ASIR, ASDR, and age-standardized DALYs rate for OUD are projected to rise from 196.63, 23.92, 0.68, and 109.68 per 100,000 people in 2021 to 339.46, 45.44, 0.94, and 150.08 per 100,000 people in 2050, respectively (Figures 7A–D). For males, the predicted ASPR, ASIR, ASDR, and age-standardized DALYs rate for OUD in 2050 are 239.62, 31.98, 2.42, and 206.44 per 100,000 people, respectively (Figures 7E–H).

## 4 Discussion

This study used the GBD 2021 database to assess the global, regional patterns and trends in the burden of OUD over a 31-year period and found that the global burden of OUD in terms of ASPR, ASDR, and age-standardized DALYs rate showed an upward trend, while the burden of OUD in terms of ASIR showed a downward trend, and the future prediction results showed that the global burden of OUD would show an upward trend in both males and females. Our results indicated the huge global burden of OUD, and active prevention and intervention measures need to be taken to reduce the burden of OUD.

Among the 21 GBD regions, the burden of OUD in High-income North America increased the fastest from 1990 to 2021, while that in East Asia decreased the fastest. This discrepancy is closely related to the implementation effectiveness of different prevention levels in the two types of regions. For primordial prevention, East Asian regions have reduced drug availability at the source through strict regulation of the production, distribution, and use of opioids (such as classified control and approval supervision systems) (13–15). In contrast, the over-marketing of prescription opioids and the proliferation of illicit opioids (e.g., fentanyl) in High-income North America have significantly increased exposure risks (16–19). For primary prevention (reducing demand): East Asia’s public health education system emphasizes the harms of substance abuse (20), whereas High-income North America lacked large-scale public awareness campaigns in the early stages. It was not until the 2000s, when the Centers for Disease Control and Prevention (CDC) updated its pain management guidelines (emphasizing the priority of non-opioid medications), that such efforts were gradually strengthened (21). For secondary prevention (early screening and intervention): High-income North America has insufficient early screening for high-risk occupational groups (e.g., construction and machinery workers) (22), while East Asia identifies at-risk individuals earlier through occupational health monitoring and community-based screening. For tertiary prevention (treatment and rehabilitation): East Asia has established a comprehensive continuum of drug detoxification treatment (e.g., detoxification centers integrating medication, psychological support, and rehabilitation training) (23, 24). Although High-income North America has emergency measures such as naloxone (e.g., the Take-home Naloxone program), it faces low accessibility and continuity of treatment (25).

This study also found that the burden of OUD in males was higher than that in females. Studies have shown that males are more likely to escalate to high-dose treatment when starting opioid therapy for chronic non-cancer pain and are more likely to die from opioid poisoning (26). In terms of occupation, males were more engaged in high-risk industries such as construction, material handling, processing, and machinery, and the proportion of people in these occupational groups who use opioids due to work-related injuries is also higher (27). This study found that the burden of OUD was heavier in young people, especially in the 20–24 age group. The risk of OUD usually begins in adolescence and young adulthood, with two-thirds of people receiving treatment for OUD reporting that their first use was before the age of 25, and one-third reporting that it was before the age of 18 (28). Notably, older adult males also faced a relatively high risk. This is mainly associated with age-related physiological changes, including the decline in liver and kidney function, which slows down drug metabolism and reduces clearance rate—making them more prone to drug accumulation and poisoning (29, 30). Meanwhile, older adult males often suffer from multiple chronic diseases (such as chronic obstructive pulmonary disease and cardiovascular diseases), and the side effects of opioids (e.g., respiratory depression and sedation) can have a synergistic effect with these underlying conditions, significantly increasing the risks of falls, fractures, and mortality (29). In addition, polypharmacy is extremely common among older adult males; the interaction between opioids and benzodiazepines or other central nervous system depressants further amplifies the risks of overdose and accidental injury (30). This phenomenon urgently highlights the need for targeted intervention measures and early intervention strategies.

The results of the decomposition analysis showed that population increase and epidemiological changes contributed more to the increase in the burden of OUD in global. Population growth not only affects the absolute number of OUD but may also indirectly affect the epidemic trend of OUD by changing the population structure and age distribution (31). The growth of the global population has directly expanded the size of the “potential at-risk population.” The increase in total population will lead to a rise in the absolute number of OUD cases, thereby pushing up the overall disease burden. The epidemiological changes involved multi-dimensional factors: regarding the evolution of opioid prescribing practices, since the 1990s, some high-income countries (particularly in North America) have had relatively loose regulations on the prescription of opioid analgesics, which led to the widespread circulation of opioids for medical use and laid hidden risks for addiction and abuse (32); although multiple guidelines (e.g., the U.S. CDC’s Guidelines for Prescribing Opioids for Chronic Pain) tightened prescribing policies after the 2000s, the dependency issues accumulated in the past continue to exert an impact (33). In terms of the expansion of the illicit opioid market, the proportion of illicitly synthesized opioids (e.g., fentanyl) in the global drug market has increased significantly in recent years (34); such drugs are highly potent and low-cost, and are often mixed into other drugs, resulting in a sharp rise in the risks of accidental overdose and addiction (35). As for regional disparities and healthcare accessibility, regions with the high SDI regions bear the heaviest burden of OUD due to historical prescribing practices and relatively high drug accessibility, while regions with a low-to-middle SDI show a rapid growth trend in OUD, driven by the infiltration of the illicit market and insufficient treatment resources (35).

Based on the APC analysis, the age effect presents a bimodal pattern, with a rise in the 20–30 age group, a decline between 30 and 80 years old, and a subsequent increase again in those aged over 80. The high risk among the 20–30 years-old young population is associated with risk-taking behaviors, social exposure, and neurodevelopmental sensitivity; the declining risk in the middle-aged to older adult group (30–80 years old) may reflect the role of clinical interventions (such as methadone maintenance treatment) and natural withdrawal. In contrast, the resurgence of risk in people over 80 is linked to age-related needs for chronic pain management, polypharmacy, and cognitive decline—factors that may lead to misuse and dose accumulation (35). The period RR of global OUD prevalence, incidence, and DALY rate all showed a downward trend. This is inseparable from the strengthened global regulation on the use of opioids. In 2000, the U.S. CDC updated its guidelines for the treatment of chronic pain, emphasizing non-opioid drugs as the first-line treatment and putting forward stricter restrictions on the use of opioids (21). Naloxone counteracts the pharmacological and toxic effects of opioids by blocking the binding of opioids to receptors and is a key drug in addressing opioid-related risks. Take-home naloxone (THN) originated in 1991 and began to be implemented at the state or national level after 2000 (36). In terms of public education, countries have also begun to strengthen the publicity and education on the problem of opioid abuse, which has a positive impact on the public’s perception of the opioid crisis and promotes a deeper understanding of the problem (37).

There are several limitations that need to be discussed. First, this study was based on GBD 2021, which is not derived from raw data, but uses a variety of mathematical models and combines a large amount of data to make predictions about the burden of disease, so the results may be biased, and second, although GBD 2021 covers 204 countries and regions, variations in data availability and quality across regions may affect the accuracy of the analysis. Particularly in areas with low SDI, insufficient medical resources and limitations in data collection may lead to underestimation of OUD cases, final, when predicting future burdens, the study failed to consider the impact of other factors.

## 5 Conclusion

Our study emphasized that the burden of OUD is on an upward trend, both from 1990 to 2021 and projected to 2050. At the regional level, the burden was most severe in high-income North America. In addition, the burden of OUD was relatively heavy among young populations and male populations. Population growth and epidemiological changes contributed significantly to the increase in the burden of OUD. These findings underscore the urgency of formulating targeted public health strategies to mitigate the escalating OUD burden.

## Data Availability

Publicly available datasets were analyzed in this study. This data can be found at: https://ghdx.healthdata.org/gbd-2021.

## Footnotes

^1^ https://www.unodc.org/unodc/en/data-and-analysis/world-drug-report-2023.html

^2^ https://ghdx.healthdata.org/gbd-2021

^3^ https://ghdx.healthdata.org/record/global-burden-disease-study-2021-gbd-2021-socio-demographic-index-sdi-1950%E2%80%932021
