# Supplementary material for: Global and regional burdens of opioid use disorder from 1990 to 2021, with future forecasts to 2050: a systematic analysis for the global burden of disease study 2021
Source: Front Public Health. 2025 Nov 20;13:1682094. doi: 10.3389/fpubh.2025.1682094 (PMC12675459; doi:10.3389/fpubh.2025.1682094)
Supplement: Supplementary file 1 [file Table_1.docx]

**Supplementary Table 1 Decomposition analysis**

| Location name | Overall difference | Aging | Population | Epidemiological change | A percent | P percent | R percent |
| --- | --- | --- | --- | --- | --- | --- | --- |
| Prevalence |  |  |  |  |  |  |  |
| Low SDI | 1052967 | 25175.55 | 966938.4 | 60852.81 | 2.39 | 91.83 | 5.78 |
| Global | 14326644 | 184334 | 8845253 | 5297057 | 1.29 | 61.74 | 36.97 |
| High SDI | 11530099 | 84702.49 | 1823355 | 9622041 | 0.73 | 15.81 | 83.45 |
| High-middle SDI | -386057 | -88989.1 | 817495.5 | -1114564 | 23.05 | -211.75 | 288.7 |
| Low-middle SDI | 1705324 | -194497 | 1693221 | 206600.2 | -11.41 | 99.29 | 12.12 |
| Middle SDI | -275769 | -46250.7 | 1972764 | -2202282 | 16.77 | -715.37 | 798.6 |
| Incidence |  |  |  |  |  |  |  |
| Low SDI | 171839.2 | -1050.79 | 177109.2 | -4219.16 | -0.61 | 103.07 | -2.46 |
| Global | 1280665 | -14110 | 1170184 | 124591 | -1.1 | 91.37 | 9.73 |
| High SDI | 835799.1 | 40000.48 | 153433.4 | 642365.2 | 4.79 | 18.36 | 76.86 |
| High-middle SDI | -18458.2 | 247.16 | 126699.5 | -145405 | -1.34 | -686.41 | 787.75 |
| Low-middle SDI | 333749 | -25565.6 | 279118.6 | 80195.93 | -7.66 | 83.63 | 24.03 |
| Middle SDI | 71404.08 | -26155.4 | 317292.3 | -219733 | -36.63 | 444.36 | -307.73 |
| Death |  |  |  |  |  |  |  |
| Low SDI | 6565 | 354.411 | 5287.263 | 923.33 | 5.4 | 80.54 | 14.06 |
| Global | 119070.2 | 5035.301 | 61755.05 | 52279.89 | 4.23 | 51.86 | 43.91 |
| High SDI | 108746.9 | 795.657 | 14463.37 | 93487.82 | 0.73 | 13.3 | 85.97 |
| High-middle SDI | -4813.22 | -384.213 | 5350.059 | -9779.07 | 7.98 | -111.15 | 203.17 |
| Low-middle SDI | 10954.64 | 535.146 | 9278.658 | 1140.837 | 4.89 | 84.7 | 10.41 |
| Middle SDI | -26183.6 | 252.165 | 15816.46 | -42252.2 | -0.96 | -60.41 | 161.37 |
| DALYs |  |  |  |  |  |  |  |
| Low SDI | 686511.2 | 20107.97 | 592668.6 | 73734.62 | 2.93 | 86.33 | 10.74 |
| Global | 10445307 | 159780.6 | 6060610 | 4224917 | 1.53 | 58.02 | 40.45 |
| High SDI | 9476872 | 183796.8 | 1360200 | 7932875 | 1.94 | 14.35 | 83.71 |
| High-middle SDI | -412416 | -9272.99 | 514986.1 | -918129 | 2.25 | -124.87 | 222.62 |
| Low-middle SDI | 1040777 | -95631.7 | 986809.6 | 149598.6 | -9.19 | 94.81 | 14.37 |
| Middle SDI | -1030296 | -70765.2 | 1413218 | -2372749 | 6.87 | -137.17 | 230.3 |
